# Identification and Characterization of Glycine- and Arginine-Rich Motifs in Proteins by a Novel GAR Motif Finder Program

**DOI:** 10.3390/genes14020330

**Published:** 2023-01-27

**Authors:** Yi-Chun Wang, Shang-Hsuan Huang, Chien-Ping Chang, Chuan Li

**Affiliations:** 1 Department of Biomedical Sciences, Chung Shan Medical University, Taichung 40201, Taiwan; 2 Department of Medical Research, Chung Shan Medical University Hospital, Taichung 40201, Taiwan

**Keywords:** fibrillarin, GAR1, glycine- and arginine-rich (GAR) motifs, GAR motif finder (GMF), RG/RGG repeat-containing proteins, arginine methylation, LLPS

## Abstract

Glycine- and arginine-rich (GAR) motifs with different combinations of RG/RGG repeats are present in many proteins. The nucleolar rRNA 2′-O-methyltransferase fibrillarin (FBL) contains a conserved long N-terminal GAR domain with more than 10 RGG plus RG repeats separated by specific amino acids, mostly phenylanalines. We developed a GAR motif finder (GMF) program based on the features of the GAR domain of FBL. The G(0,3)-X(0,1)-R-G(1,2)-X(0,5)-G(0,2)-X(0,1)-R-G(1,2) pattern allows the accommodation of extra-long GAR motifs with continuous RG/RGG interrupted by polyglycine or other amino acids. The program has a graphic interface and can easily output the results as .csv and .txt files. We used GMF to show the characteristics of the long GAR domains in FBL and two other nucleolar proteins, nucleolin and GAR1. GMF analyses can illustrate the similarities and also differences between the long GAR domains in the three nucleolar proteins and motifs in other typical RG/RGG-repeat-containing proteins, specifically the FET family members FUS, EWS, and TAF15 in position, motif length, RG/RGG number, and amino acid composition. We also used GMF to analyze the human proteome and focused on the ones with at least 10 RGG plus RG repeats. We showed the classification of the long GAR motifs and their putative correlation with protein/RNA interactions and liquid–liquid phase separation. The GMF algorithm can facilitate further systematic analyses of the GAR motifs in proteins and proteomes.

## 1. Introduction

Eukaryotic protein-coding genes may acquire some novel features to accommodate the encoded proteins in the enormously large and complicated cellular environment. Fibrillarin (FBL), an rRNA 2′-O-methyltransferase (MTase), has a long N-terminal GAR domain with diverse combinations of arginine–glycine–glycine (RGG) or arginine–glycine (RG) repeats in eukaryotes but not Archae [1]. On the other hand, the C-terminal MTase domain of FBL is highly conserved in archaea and eukaryotes. FBL is an abundant nucleolar rRNA 2′-O-methyltransferase (MTase) using the guide box C/D small nucleolar RNAs (snoRNAs) to recognize the target sites in rRNA [2]. A nucleolar localization signal appears to be present in the GAR domain of human FBL [3]. Arginine methylation in the GAR domain can regulate the nuclear and nucleolar localization of FBL [1]. Thus, long GAR domains in eukaryotic FBL might be at least partially correlated with novel sub-compartmentation requirements in eukaryotic cells. Due to its critical roles in ribosome formation and protein synthesis, FBL is involved in tumorigenesis and viral infections and can be considered as a therapeutic target [4,5].

Membrane-less organelles (MLOs) or numerous RNP bodies organize and function through the liquid–liquid phase separation (LLPS) mechanism to coordinate thousands of simultaneous molecular reactions spatiotemporally [6]. Nucleoli, the most prominent MLO in cells for rRNA synthesis, processing, and ribosome assembly, are liquid-like-phase-immiscible with the neighboring nucleoplasm. Ribosomal RNAs are transcribed at the border of fibrillar center (FC) and dense fibrillar component (DFC), processed first at DFC and then at the granular component (GC), the most outer layer of the nucleolus. The GAR domain appears to be critical for phase separation of FBL in nucleoli. Purified GAR domain is sufficient for phase separation, but the MTase domain or full-length FBL confers immiscibility with GC components [6]. The GAR domain of FBL can self-associate to promote nascent 47S pre-rRNA sorting and processing for the assembly of DFC through phase separation [7].

In addition to FBL, two other nucleolar proteins, nucleolin (NCL or C23) and GAR1 (NOLA1; H/ACA ribonucleoprotein complex subunit 1), also contain long GAR domains [8]. Human NCL is a nucleolar phosphoprotein with long acidic Asp/Glu-rich segments in the N-terminal half and a GAR domain at the C-terminus. GAR1, a component of the H/ACA small nucleolar ribonucleoprotein (box H/ACA snoRNP) complex catalyzing the pseudouridylation of rRNA, has one GAR domain at the N-terminus and one other at the C-terminus [9]. The GAR domains in the three nucleolar proteins are usually longer than 50 amino acids. FBL and GAR1 are both DFC components, while NCL is a typical GC marker.

Early biochemical analyses identified multiple asymmetric *N^G^, N^G^*-dimethylarginines (ADMA) surrounded by glycine residues in FBL and NCL, indicating post-translational modification of arginines in GAR domains [10,11]. Arginine methylation in the GAR domain of GAR1 was also identified [12,13]. “RGG box”, based on the tri-RGG sequence conserved in hnRNPU that binds RNA homopolymers, is another term for GAR motifs [14]. Many RGG box-containing hnRNP proteins also contain ADMA [15]. Protein arginine methyltransferases (PRMTs), widely distributed in eukaryotes, are responsible for the modification. There are nine different PRMTs identified in humans. Type I PRMTs, including PRMT1, 2, 3, 4 (or CARM1), and 6, transfer two methyl groups on different guanidino nitrogens of the arginine residues to form the ADMAs in the nucleolar GAR-domain-containing proteins and hnRNPs mentioned above. Type II PRMTs (PRMT5 and 9) transfer two methyl groups on the same guanidino nitrogen to form symmetric dimethylarginines (SDMA), mostly in proteins with RG repeats. Both type I and type II PRMTs catalyze the formation of monomethylarginines (MMA) first, and then, from this intermediate, type I PRMTs catalyze the formation of ADMA, whereas type II PRMTs catalyze the formation of SDMA. PRMT7 is the single type III enzyme that only puts a methyl group on the arginine residues to form monomethylarginines (MMA) [16,17]. Arginine methylation might crosstalk with other post-translational modifications. For example, the SRGG repeats in the GAR motif of yeast FBL (Nop1p) can be modified by arginine methylation as well as serine phosphorylation and dephosphorylation. Negative charged phosphoserines, such as aspartates, can block methylation of neighboring arginine residues [18]. Furthermore, many GAR-motif-containing proteins exist in numerous RNPs or MLOs, and arginine methylation of the motifs can modulate these proteins participating in LLPS for MLO assembly [19].

Bioinformatics tools for systematic analyses of the GAR motifs in proteins should facilitate related research. Previous systematic analysis of human proteome classified proteins as tri-RGG, di-RGG, tri-RG, and di-RG according to the presence of RGG or RG repeats with a gap of 0–4 amino acids [8]. However, the classification might be confusing as many GAR-motif-containing proteins have both RG and RGG repeats. Moreover, no bioinformatics tools have been designed to identify and characterize these motifs in proteins conveniently. We have studied protein arginine methylation of FBL [20,21] and two other GAR-motif-containing nucleic acid proteins: SERBP1 [22,23] and CNBP [24]. We are aware that though various proteins, especially many nucleic acid binding proteins, have RGG box sequences, only a few contain a GAR domain with continuous RG/RGG repeats of more than 10 times and longer than 50 amino acids, such as FBL. Typically, “domain” indicates the independently folded protein structure with a specific function, and “motif” designates combinations of short neighboring segments of secondary structures. In this study, we usually refer to extra-long GR/RGG-repeat-containing sequences for more than 50 amino acid residues as GAR domains and combinations of RG/RGG repeats within a limited length as GAR motifs. We are interested in the long GAR domain in FBL through evolution and the existence of similar long GAR domains in other proteins. We also would like to characterize the GAR motifs and domains in different proteins. We thus developed a GAR MOTIF FINDER (GMF) program to identify the motifs in proteins and further demonstrate the characteristics of GAR motifs. By GMF, we showed our analyses of the GAR domain of FBL and the other two nucleolar proteins as well as the GAR motifs in other typical known RGG/RG-containing proteins. We then analyzed the whole human proteome for all the long GAR-motif-containing proteins.

## 2. Materials and Methods

### Program Coding of GAR Motif Finder (GMF)

This program was written in Python with the graphic user interface (GUI) constructed by tkinter, and matplotlib integrated into the GUI to show the pie chart. It is available at https://mega.nz/file/icYyiRzT#IKknEik5PojpaxoK23zMKEXRXIglAGKN9IUCeXqAjPY (accessed on 16 January 2023) for download. The GMF program allows users to examine and locate the GAR motifs in target protein sequences. The pattern G(0,3)-X(0,1)-R-G(1,2) -G(0,3)-X(0,5)-G(0,3)-X(0,1)-R-G(1,2) is employed to find the GAR motif with multiple RG/RGGs that might be interrupted by long flexible G-rich tracts or some other amino acids. X indicates any amino acid residue. The numbers in the parentheses separated by a comma are the minimum and maximum times the residues can repeat at the position. The input sequences must be in the FASTA format. Single or multiple sequences can be analyzed each time. The text results shown in the information window include the accession numbers (names) of the input sequences, the position of the motif in the sequence, the motif sequence (pattern), the numbers of RG or RGG repeats, the non-G or R (else) amino acids in the motifs, the percentage coverage of the GAR motif in the polypeptide, the G to R ratios, the percentages of G, R, and other amino acids in the motifs, and the complete entry sequences with GAR motifs bracketed. If more than one sequence is in the input, at the end of the text, the total statistics show the number of input sequences, the number of sequences with GAR motifs, the percentage of the input entries with GAR motifs, and the number of total RG, RGG, and non-GR amino acids in the motifs. The results can be exported as a .txt file or can output as a table containing the key information as a .csv file. In addition, the graph window can show the pie chart of the RG/RGG percentage as well as the bar graph of the amino acids other than R and G in all the GAR motifs of the input sequences.

## 3. Results

### 3.1. Analyses of the N-Terminal GAR Domain of Fibrillarin in Different Model Organisms

To develop an algorithm to analyze the GAR motifs, we need to characterize the critical parameters of the motif first. The GAR domain at the N-terminus of FBL has been well studied and should be an excellent example to extract the key information. We retrieved and analyzed the FBL sequences from evolutionarily distant model organisms: yeast (*Saccharomyces cerevisiae*), nematodes (*Caenorhabditis elegans*), and fruit flies (*Drosophila melanogaster*). We also analyzed FBL from five vertebrate species, including a bony fish (zebrafish, *Danio rerio*), an amphibian (clawed frog, *Xenopus tropicalis*), a reptile (green anole, *Anolis carolinensis*), and two mammals (mouse, *Mus musculus*; humans, *Homo sapiens*) for conservations and variation in a specific lineage. We loosely defined the GAR domain from the first RGG to the last RG(G) sequence before the conserved EPHR sequences. The alignment is shown in Supplementary Figure S1.

To show the key features of these domains, we calculated and summarized in Table 1 the lengths of the GAR domain/the full-length FBL, G/R ratios, and percentage of G (G%), R (R%), and non-GR amino acids (non-RG%) in the domains. The lengths of the domains vary from 72 to 107 in the FBL orthologues. Other amino acids might be directly before but are not directly after R in the domain. The G/R ratios vary from 2.47 (yeast) to 4.73 (fruit fly), mostly around 3. Thus, most of the Rs in the domain are followed by two Gs as RGG. All Rs in the GAR domains are followed by at least one G as RG_(1–13)_. The R% within the domain is usually around 20%, while the G% varies greatly in different FBL orthologues. Fly FBL contains five RG_4_ and RG_5, 6, 7, 10, 12_, resulting in the highest G% (77.2%). Table 1 also lists the numbers and percentages of the non-GR amino acids. Other intervening amino acids are different between species, but phenylalanine (F) is most frequently detected. The listed features in Table 1 reflect the variances and similarities of these domains.

### 3.2. Development of the GAR MOTIF FINDER Program for Analyses of Long GAR Domain in FBL

Though we analyzed the GAR domain in the FBL proteins manually in the previous section, it is inefficient to conduct similar analyses to more target sequences. We then tried to develop an algorithm GAR MOTIF FINDER (GMF) to search for the motifs containing repetitive RG or RGG elements and conducted some pilot analyses with the FBL sequences. After adjustments with different GAR motif pattern combinations, especially to include most of the extra-long polyglycine (polyG) sequences and short non-RG segments in some GAR domains of FBL, we defined the GAR motif as G(0,3)-X(0,1)-R-G(1,2) -G(0,3)-X(0,5)-G(0,3)-X(0,1)-R-G(1,2). This pattern limits the identified motif to have at least two elements of either RG or RGG and allows the flexibility with polyG tracts (up to 13 Gs in straight) between the arginine residues. It can also accommodate at most six other intervening amino acid residues between RG/RGG elements. The X(0,1)-R-G(1,2) module considers the multiple FRGG, SRGG, ARGG, DRGG, or PRGG motifs that frequently occur in the GAR domain of FBL.

**Figure 1:**
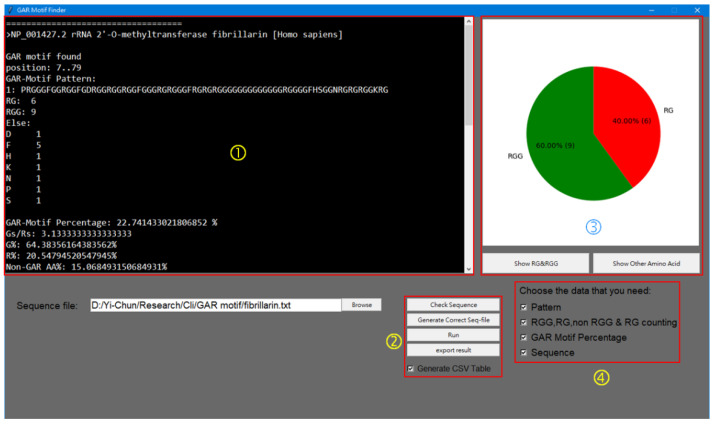
The interface of GMF. Red box ➀ is the information window to show the events of any operation. Red box ➁ is the function button panel. Red box ➂ is the graphic window to display the pie or bar charts. Red box ➃ is the check box to select the data to be analyzed.

Figure 1 shows the interface window of GMF. Users can input the sequences to be analyzed singly or in batches by uploading the .txt files with the sequences to be analyzed in FASTA format. Text results are shown in the window or can be exported to the same folder of the sequence file. The result can also be exported as an excel table (.csv file). The outputs, whether in text or in table, include features in Table 1 plus the sequence pattern of the motif, the position (numbers of the start and end residues) of the motif, the percentage of the motif in the full-length polypeptide, and the numbers of RG or RGG repeats in order to provide more information to evaluate the motif.

### 3.3. Analyses of the GAR Domains in the Three Nucleolar Proteins by GMF

In addition to FBL, two nucleolar proteins, NCL and GAR1, also have long GAR domains of about 50 amino acids and are classified as tri-RGG proteins by Thandapani et al. [8]. To show the power of the program to analyze multiple sequences, we retrieved sequences of the three nucleolar proteins FBL, NCL, and GAR1 from five different vertebrate model organisms, including zebrafish, clawed frog, green anole, mouse, and humans, to characterize their GAR domains by GMF. Features of the GAR domains in these proteins (vFNG) from the GMF analyses are shown in Table 2 (direct .csv output by GMF in Supplementary Table S1 and .txt output in Supplementary File S1). Minor differences in the FBL features in Table 1 and Table 2 should be due to slightly different domain definitions. The GAR domains in the vFNG proteins generally are long with more than 10 RGG plus 0–4 RG elements. The GAR domains of FBL are longer than that of NCL or GAR1 and contain the highest repeat numbers of RGG plus RG. The percentages of the motifs are about 20–24% for FBL, 6–8% for NCL, and 22–28% for the N-terminal as well as the C-terminal GAR domains of GAR1, reflecting the differences in the full lengths of these proteins. Of the 15 vFNG entries, only the GAR domain in zebrafish FBL is separated into two GAR motifs (residues 7–38 and 50–79) by the defined pattern of GMF. As the identified motifs are bracketed in the full-length sequence at the end of the entry in the text report, the interrupted G-rich sequence FGGGFKSPGGE between the two motifs can be easily identified. There is one more short ERGGGGRG motif identified by GMF in zebrafish NCL, about 90 amino acid residues upstream of the long C-terminal GAR domain. There is only one RGG element at similar positions in other NCL sequences, thus it would not be indicated by GMF.

As shown in Table 2, the G/R ratios in these GAR domains are from 2.12 to 4.38, mostly over 2.5, indicating that usually there are Gs between the RGG/RG repeats. The non-GR% are generally below 20%. The GAR domains of NCL orthologues have the highest purity of “RGG” as they consist of 9–12 RGGs but no or only one RG. Furthermore, the GAR domains of NCL have high G/R percentages, and F is almost the only intervening amino acid. The two GAR domains of GAR1 account for about 50% of this short polypeptide. It is interesting that from fish to amphibian, reptiles, and mammals, the N-terminal GAR (GAR-N) domains become longer and more diverse than the C-terminal ones. On the contrary, the C-terminal GAR domains (GAR-C) become shorter and contain only RG_(1–6)_ repeats interrupted by F. Among all GAR domains of vFNG, the GAR-N domains of GAR1 in green anole, mouse, and humans, the three amniotic species, have the highest percentage (~23–24%, mostly F, N, P, and S), while the GAR-C domains contain the lowest (7–8%) percentage of non-GR amino acids.

**Figure 2:**
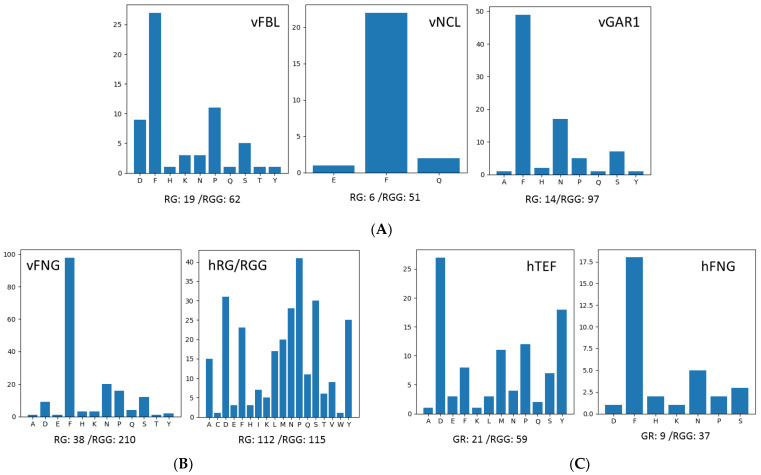
The distribution pattern of non-GR amino acids in the GAR domains. GMF output of the distribution of non-GR amino acids in the GAR domains of specific protein groups. The total numbers of RG or RGG repeats in the GAR domains of the protein groups are shown under the bar graph. (**A**) The distribution pattern of non-GR amino acids in the GAR domain of 3 nucleolar protein FBL, NCL, and GAR1 from 5 vertebrate model organisms (FBL, NCL, and GAR1). (**B**) The non-GR amino acids in the GAR domains of the three nucleolar proteins from the 5 model vertebrate species (vFNG) and that in the GAR motifs of 16 human RG/RGG-repeat-containing proteins (hRG/RGG). (**C**) The non-GR amino acids in the GAR domains of human FBL together with NCL and GAR1 (hFNG) and that of the human FET family members (hFET).

The majorities of the Rs in these GAR domains of vFNG are as RGG but not RG (in total 210 RGG/38 RG). Besides showing the identities and numbers of non-GR amino acids of each GAR motif, GMF can sum and output the distribution of non-GR amino acids in all GAR motifs identified in the search as bar graphs. We input vFBL, vNCL, and vGAR1 separately and the overall non-GR amino acid distributions are as shown in Figure 2A. F is the most abundant one in these domains. FBL orthologues have the longest GAR (with 19 RG/62 RGG and 27 F) and the most diverse composition with 10 other interspersed amino acids in total. The GAR domains of NCL basically are composed of only G, R, and F (with 5 RG/51 RGG and 22 F). The two GAR domains of GAR1 orthologues (with 14 RG/97 RGG, 49 F) have eight non-GR amino acids. The degree of variations of the length, RGG/RG distribution, or the percentage of G, R, and non-GR amino acids of the GAR domains of any FNG protein orthologues in vertebrate species basically are correlated to their evolutionary distances.

### 3.4. Analyses of Other High RG/RGG-Repeat-Containing Proteins by GMF

As shown above, all three nucleolar proteins contain more RGG than RG repeats and have multiple Fs. We were interested whether GAR motifs in other proteins also share these features. We used GMF to analyze 17 human proteins in the list of RG/RGG-repeat-containing (hRG/RGG) proteins of a previous study [25] and compared the results with those of the three nucleolar proteins.

Interestingly, though multiple RG or RGG elements are present in hnRNPA2/B1, they are discontinuous and cannot be defined as GAR motifs by GMF. GMF analyses of the other 16 proteins are shown in Table 3 (direct .csv and .txt output by GMF in Supplementary Table S2 and Supplementary File S2). In total, 43 GAR motifs are identified in these proteins. There might be more than one GAR motif in one protein and the motifs might scatter throughout the polypeptides. Most motifs in some proteins such as the FET family members (FUS, EWS, and TAF15) and Lsm14a (RAP55A) are RGG-rich, while motifs in other proteins such as KHDR1 (Sam68), caprin-1, and kmt2b are RG-rich. Other RNA-binding proteins in the list such as SERBP1, hnRNPA1, hnRNPU, DDX4, G3BP1, FMR1, and FXR1 contain motifs with RG and RGG of similar levels. Moreover, different motifs in any single protein might be RG- or RGG-rich. For example, the first GAR motif in SERBP1 has 6 RGs/2 RGGs, but the second one contains 2 RGs/4 RGGs. The total RG/RGG numbers of all GAR motifs in these 16 proteins are 112/115, and 9 of the 16 proteins were classified as tri-RGG, 3 as di-RGG (FMRP, FXR1, and Sam68), 2 as tri-RG (DDX4 and Caprin-1), and 1 as di-RG (G3BP1) by Thandapani et al. [8], which are shown by the color codes in Table 3. The G/R ratios in different motifs range from 1.0 to 4.5. The non-GR % range from 0 to more than 50%. Proline (P) is the most abundant non-RG amino acid after summing up all GAR motifs in these hRG/RGG proteins, and D, S, N, and Y are also widely distributed (Figure 2B). F is not the most abundant non-GR amino acid in the GAR motifs of these proteins.

For the GAR motifs listed in Table 3, few are longer than 50 amino acids with more than 10 RG plus RGG repeats, such as the long GAR domains in FNG. Specifically, the longest or the fourth GAR motif in EWS and TAF15 contain 2RG/10RGG (58 residues) and 11 RGG repeats (82 residues), respectively. The longest GAR motif in CHTOP is of 53 residues with 10 RG/5 RGG, and in Lsm14a, it is of 45 amino acids with 4 RG/8 RGG. Within them, the long GAR domains of EWS and Lsm14a are close to the ones in FNG, with multiple RGG but few RG, several Fs in the motifs, and a non-GR % of about 24%.

The FET family proteins are structurally related but functionally different RNA binding proteins [26]. They all contain SYGQ -rich sequences at the N-terminus and separated GAR motifs of various lengths after this region. GMF identified five GAR motifs in EWS and TAF15 and four in FUS, distributed from the middle to close to the C-terminus of the polypeptides. There are repetitive PGG elements between GAR motif 2 and 3 and also motif 4 and 5, in EWS. The longest GAR, motif 4 in TAF15, is not disrupted by such elements but contains 10 GGYGGD repeats between the RGGs, making this motif much longer than the longest motif in FUS and EWS. GAR motif 4, the longest one in FUS with 34-amino acids consisting eight RGGs and one RG, is shorter than those in EWS and TAF15.

The overall RG/RGG repeat numbers of hFET are 21/59. The RG/RGG numbers of the longest motifs in these proteins are 3/29, indicating the longest motifs have strong RGG preferences. Though the high RGG preference is similar to hFNG (9 RG and 37 RGG in total), the distribution patterns of non-GR amino acids in the GAR motifs in each group are distinctive. While FNG all contain multiple Fs, F is not the major non-GR amino acid in any single motif of the FET proteins. The longest motif in TAF15 has 10 D(S)RGG(G) YGG repeats with 11 Ds and 10 Ys, making D the most frequent non-GR amino acid in hFET followed by Y (Figure 2C). D also locates in some short GAR motifs in the FET proteins, but Y is restrictively distributed in TAF15. On the other hand, P and M are frequently encountered in EWS. Therefore, the longest GAR domains in FET proteins are different from those of the three nucleolar proteins. They are even variable within the FET group, though they all are long and with repetitive RGGs.

### 3.5. Analyses of Extra-Long GAR Motifs in Human and Other Proteomes

As the GMF program was coded for identification and characterization of extra-long GAR motifs in proteins, we then used GMF to analyze the human proteome (*GRCh38*.*p13* from Genome Reference Consortium) for all extra-long GAR domains. Many proteins in the human proteome are isoforms due to alternative splicing and some GMF hits might have dozens of isoforms. We sorted and inspected the GMF results in the .csv file and identified 21 motifs in 161 protein isoforms encoded by 18 genes with the RG/RGG repeat numbers higher than 10. A summary of the GMF analyses of the 21 long GAR motifs is shown in Table 4, and the numbers of isoforms containing the motifs are indicated (.csv GMF output of the isoforms and all motifs in the proteins are in Supplementary Table S3). We found that in the human proteome, the number of RG plus RGG repeats in one single GAR motif is 16 (11 RG plus 5 RGG repeats) at most. Two paralogous proteins, heterogeneous nuclear ribonucleoprotein Q (hnRNPQ) and hnRNPR, contain such motifs. Interestingly, different isoforms of hnRNPQ have variations at the C-terminus of the longest GAR domain, resulting in 11/5, 10/3, and 9/4 of the RG/RGG repeat numbers.

Besides hnRNP R and Q, there are 10 other proteins containing more RG than RGG repeats. Zinc finger CCCH domain-containing protein 4 (ZC3H4) is the only protein with two motifs that have RG/RGG repeats of more than 10. The first motif contains 13 RGs and 1 RGG and the second one has 9 RGs and 2 RGGs. Methyl-CpG-binding domain protein 2 (MBD2) has a GAR motif with 12 RGs and 2 RGGs. Though the percentages of G, R, and non-GR amino acids of the three motifs are similar, the identity of non-RG amino acids between proteins vary greatly. Myosin XVB contains 5 GAR motifs and the longest one has 11 RG repeats. Some target proteins such as myosin XVB and Bromodomain and WD-repeat-containing protein 3 (BRWD3) (9 RGs and 4 RGGs) are very long, with the longest GAR domain accounting for only about 2% of the protein. The long motifs in RNA-binding protein 26 (RPM26) and histone-lysine N-methyltransferase EHMT2 both account for about 3% of the protein, containing 10 RGs and 9 RGs plus 1 RGG, respectively. On the contrary, the GAR domain with 10 RG and 5 RGG repeats of the chromatin target of PRMT1 protein (CHTOP, also shown in Table 3) accounts for more than one-fifth of the protein. The GAR motif may simply contain only RG repeats without any interrupting non-RG amino acids such as the one with 9 RGs and 1 RGG in zinc finger protein 579 (ZNF579). On the contrary, it may contain more complicated tandem repeats such as the GAR motif in initiation factor 3 subunit A (eIF-3A) with 5 PRRGL/(M)DDDRG repeats (11 RG repeats separated by multiple Ds). The only ribosomal protein on the list is 40S ribosomal protein S2 (RPS2) with 8 RGs and 3 RGGs.

The six proteins containing the long GAR domains with more RGG than RG repeats are the three FNG proteins EWS, TAF19, and Lsm14a that we described in previous sections. Therefore, long GAR domains are rare in the human proteome, especially the ones with more RGG than RG repeats.

## 4. Discussion

The rRNA 2′-O-methyltransferase FBL is a major nucleolar protein for rRNA processing. We analyzed the N-terminal GAR domains in FBL from evolutionarily distant model organisms and showed their features (Table 1 and Table 2). The long GAR domains contain more than 10 RGGs but few RG repeats connected with multiple Gs or some other amino acids, mostly F, for longer than 50 amino acids. Based on the GAR domain in different FBL orthologues, we defined the GAR motif with G(0,3)-X(0,5)-G(0,3)-X(0,1) between R-G(1,2) to allow multiple Gs surrounding the arginine residues and a few other amino acids between each RG/RGG sequences. We developed the GMF algorithm to facilitate the characterization and comparison of GAR domains containing repetitive RG or RGG elements in different proteins. Our criteria for GAR motif is more relaxed and flexible than the tri-RGG[RGG(X_0-4_)RGG(X_0-4_)RGG], di-RGG[RGG(X_0-4_)RGG], tri-RG[RG(X_0-4_)RG(X_0-4_)RG], or di-RG[RG(X_0-4_)RG] motifs defined previously [8]. This pattern is optimized to accommodate some long G arrays or a short segment of non-GR amino acids in the GAR domain in FBL. Though the main goal of the GMF program is to find and characterize long GAR motifs with repetitive RG or RGG repeats, segments with partial match of the pattern as short as four amino acid residues (RGRG) or a long motif extended over the pattern can all be identified.

GMF analyses of FBL as well as two nucleolar proteins NCL and GAR1 in five vertebrate species showed the potential of the program to help inspect the domains through evolution. In Table 2, zebrafish is the only species with the long GAR domain of FBL split in the middle, even under the relaxed GAR definition of GMF. Zebrafish NCL also has an extra short GAR motif not identified in others. Analyses of these proteins in other bony fish and vertebrate species might reveal if the differences are common in other fish. We also noticed a reverse length trend for the two GAR domains in GAR1. While the GAR-N domains become longer and more diverse, the GAR-C domain becomes shorter from fish to amniotic species, thus maintaining a balanced total length.

Besides the FNG proteins, we analyzed other typical human RG/RGG-repeat-containing proteins listed in the review by Chong et al. [25]. GMF analyses showed that GAR motifs of these proteins basically are different from the GAR domains of the nucleolar proteins. For example, the long GAR domains of the three nucleolar proteins are either at the N-terminus (FBL), the C-terminus (NCL), or both (GAR1), but other RG/RGG-repeat-containing proteins might contain one or a few GAR motifs with different lengths at various positions of the polypeptides. The features of the extra-long GAR domains of the three nucleolar proteins include numerous RGGs but few RGs, and multiple Fs but fewer other amino acids. However, the percentage of F in the GAR motifs of other RG/RGG-repeat-containing proteins is low, while P, D, and S are more frequently encountered (Table 3 and Figure 2B,C). Specifically, we compared the long GAR domains in FNG with those in FET proteins. Though they all are long with repetitive RGGs, the non-GR amino acids are different. For each FET protein, the non-GR amino acids in the long and other short GAR motifs are also different. In addition, the GAR domains of the three nucleolar proteins are either at the N-terminus (FBL) or the C-terminus (NCL), or both (GAR1), but in FET, there are 3–4 short GAR motifs distributed in the proteins and 1 long GAR domain at the C-terminal part.

Identifications of long GAR domains in NCL or FBL are connected with arginine methylation in these domains [10,11]. GAR1 is also modified by arginine methylation [12,13]. Experimental manipulation of FBL showed that no matter whether the GAR domains are randomized, truncated, or extended, they can self-associate for pre-rRNA sorting and processing, with natural GAR length optimal [7]. Swapping either of the two GAR domains from GAR1 to FBL can complement the function of FBL for pre-rRNA sorting and processing [7]. Though the long RG/RGG core in FBL, NCL, and GAR1 is conserved from evolutionarily distant species, the exact sequences or lengths are not conserved, indicating they are not critical. Maintenance of the GAR domains in these proteins thus is likely to be related to the essential modification. Methylation of the guanidino nitrogens of arginine residues reserves the positive charge, yet increases steric hindrance, and lost hydrogen bonds might lead to modified interactomes and phase separation [25]. Phenylalanine is frequently distributed in the GAR domains of FNG in the GFRG or GFG context. Due to the hydrophobicity and π electrons, phenylalanine has been used as a methylarginine mimic in some studies [27,28,29,30,31]. It is possible that in the extra-long GAR domains of the nucleolar proteins, F might function like a constitutive methylarginine in the context. Dispersed F in the GAR domains may allow a flexible range of arginine methylation levels to meet the basal requirement in these proteins. Consistent with the hypothesis, F in the GAR domain of NCL has been reported to be important for G-quadruplex binding and folding [32].

The RG/RGG-repeat-containing proteins analyzed in Table 3 are also methylated at the arginine residues in the GAR motifs [25], but P, D, and S become the major non-GR amino acids. Amino acid residues interspersed in the GAR domain can affect arginine methylation. Proteomic analyses showed that P is second to G at the +1 or −1 positions of methylarginines [33], consistent with the high frequency of P in the GAR motifs. Amino acid residues interspersed in the GAR domain can affect arginine methylation. Negatively charged amino acids such as aspartate block methylation of neighboring arginine residues [18]. Though both FUS and EWS are heavily arginine methylated, negatively charged aspartic acids neighboring R residues interfere with PRMT binding and reduce methylation in the GAR domain of another FET family member, TAF15 [26]. Similarly, phosphorylated serines mimic aspartates and can reduce neighboring arginine methylation [18]. Spontaneous deamidation of asparagine or glutamine residues can result in negatively charged residues and might also modulate arginine methylation in the domain. It is thus critical to analyze the presence of different intervening amino acids that might adjust the arginine methylation of the GAR domain.

Most of the GAR-motif-containing proteins listed in this study are components of different MLOs or RNPs. Different GAR motifs, together with other regions in the proteins, determine their subcellular distribution. Besides ionic interaction, the guanidino groups can also form π stacking and cation-π stacking. Glycine is the smallest and most flexible amino acid to tolerate a wide range of backbone arrangements. The unordered, extended, and flexible RGG/RG-rich motifs can provide multivalency for intra- or inter-molecular interactions for phase separation [25]. Arginine methylation of the GAR motifs can affect LLPS of the proteins for MLOs assemblies [19]. One of the best studied example is that of arginine methylation in the long GAR motif of FUS, which can reduce the cation-π interaction of the motif with the N-terminal low-complexity domain, preventing phase separation and further amyloid formation [34]. Arrangements of the RGG and RG repeats as well as other amino acids with different chemical properties in the GAR motifs should be critical for LLPS to determine the distribution of these proteins, and may affect the pathogenesis of neurological diseases, cancers, and viral infections.

We also explored the whole human proteome using GMF for more long GAR-motif-containing proteins. The six long GAR domains in FBL, NCL, GAR1 (GAR-C), EWS, TAF15, and Lsm14a with more RGG than RG repeats have all been analyzed in Table 2 and 3, indicating the small pool of this type of long GAR domains in the human proteome. FBL, NCL, GAR1 are nucleolar proteins. Lsm14a is a component of cytoplasmic processing bodies. EWS and TAF15 (as well as FUS) can localize to cytoplasmic stress granules and paraspeckles and pathologically accumulate as cytosolic inclusions in patients with *amyotrophic lateral sclerosis* (ALS) and frontotemporal lobar degeneration (FTLD) [35]. The long GAR domains, together with other parts in in these proteins, can lead to the LLPS of these proteins in specific MLOs. Paralogous hnRNP proteins hnRNP R and hnRNP Q both contain the GAR domain with the highest RG/RGG repeat numbers identified by GMF. Relaxed GMF motif definition results in the continuation of the domain because some RG or RGG repeats are separated by 5–6 non-GR amino acids. Arginine methylation of RGG box-containing hnRNP proteins, including hnRNP R and Q, accounts for 65% of nuclear ADMA levels [15]. Methylation of multiple arginine residues by PRMT1 in the long GAR motif of hnRNP Q has been reported [36]. Both hnRNP Q and R also accumulate in pathological inclusions in FTLD with FUS [37].

Among the 10 other proteins containing more RG than RGG repeats, 3 are nuclear proteins containing zinc fingers. ZC3H4, containing two long GAR motifs, is an RNA-binding protein localized at chromatin to suppress transcription of non-coding RNAs [38]. Both motifs are before the three continuous C3H1 zinc finger motifs at the N-terminal half of the protein. RBM26 is an RNA binding protein with one C3H1 zinc finger and two RNA binding motifs (RRM). ZNF579 has eight C2H2 zinc fingers and the GAR motif is specific in that nine RG and the final RGG repeats are without any interrupting non-RG amino acids. PRMT5 has a “GRG” substrate preference and the GAR motif in ZNF579 is modified by symmetric di-methylation [39].

A few other proteins show chromatin association and/or are related to epigenetic modification. CHTOP can bind to PRMT1, as its full name is “chromatin target of PRMT1 protein” [40], and promote methylation of arginine 3 of histone H4 (H4R3). Its binding to 5-hydroxymethylcytosine (5hmC) can help to recruit the CHTOP-methylosome complex to specific sites on the chromosome for selective gene activation [41]. Methyl-CpG-binding domain protein 2 (MBD2) is a component of the MeCP1 complex that contains HDAC1 and HDAC2 [42]. BRWD3 is a nuclear protein with eight WD repeats at the N-terminal half and two Bromo domains at the C-terminal part, and the GAR motif is near the C-terminal end. Histone-lysine N-methyltransferase EHMT2 with the GAR motif right at the N-terminus is a set domain H3K9 methyltransferase [43]. These proteins then might modulate post-translational modification (CHTOP and MBD2), or are writers to put on the modification (EHMT2), or are readers for modified bases in DNA (CHTOP and MBD2) or PTM (BRWD3). LLPS can also explain sub-chromatin structure formation and transcriptional control. For example, MBD2 can induce clustering of pericentric heterochromatin and is critical for chromocenter structure [44]. These GAR-motifs with strong RG preference might play more roles in chromatin sub-compartmentation.

Two cytosolic RG-rich proteins are involved in translation. The long GAR motif in initiation factor 3 subunit A (eIF-3A) contains 5 tandem PRRGL/(M)DDDRG repeats within a region with 25 approximate tandem repeats. This specific long GAR motif with RG repeats separated by multiple negative-charged Ds is also a mixed charge domain (MCD). MCDs with multiple RD repeats promote nuclear speckle condensation [45]. Whether and how this GAR/MCD plays in the regulation of the eIF3 complex is an interesting issue. The N-terminal long GAR motif of RPS2 is methylated by PRMT3 [46,47].

In summary, GMF can show the pattern, the position, the numbers of RG/RGG repeats, the non-GR amino acids in the motifs, the coverage, the G/R ratios, and the percentages of G, R, and other amino acids in the motifs and thus can provide critical information for further evaluation of the motifs in LLPS. The GMF program can be a starting tool to facilitate the analyses of GAR motifs in proteins through evolution as well as to design putative therapeutic targets focusing on the motifs. Further modification of the GMF program, for example, to include the analyses of the FGG/FG repeats and other elements, can improve and expand the application of the program.

## Figures and Tables

**Table 1:** The length, number of G/R residues and amino acids besides GR in the GAR domain of FBL in model organisms.

Species	GAR/Full-Length	G/R Ratio	Non-GR Amino Acids	G% ^1^	R% ^1^	non-GR% ^1^
*S. cerevisiae*	76/327	42/17 2.47	3F, 8S, 6A	55.2	22.3	22.3
*C. elegans*	107/352	62/22 2.82	7F, 5S, 5D, 3P, H, A, M	57.9	20.6	21.5
*D. melanogaster*	92/344	71/15 4.73	4F, P, A	77.2	16.3	6.5
*D. rerio*	72/317	43/16 2.68	6F, 2P, D, K, S, E, T	59.7	22.2	18.1
*H. sapiens*	72/321	47/15 3.29	5F, D, H, S, N, K	65.3	20.8	13.9
*M. musculus*	78/327	51/16 3.19	6F, Q, S, N, K	65.4	20.5	12.8
*X. laevis*	76/325	43/17 2.53	7F, 4D, 2P, Y, K, S	56.6	22.4	21.1
*A. carolinensis*	66/311	36/17 2.11	5F, 3P, 2D, 2S, N	54.5	25.8	19.7

^1^ G%, R%, and non-GR% are the percentages of G, R, and non-GR amino acids in the GAR domain.

**Table 2:** Analyses of the GAR motifs in FBL, NCL, and GAR1 in five model vertebrate species by GMF.

Accession Number	Pattern	Position	RG	RGG	Else	Total%	G/R	G%	R%	Non- GR%
FBL										
>NP_998167. [Danio rerio]	PRGGGGRGGFGGRGRGGGDRGGRGGFRGGRGG	7..38	1	7	‘P’: 1, ‘F’: 2, ‘D’: 1	10.1	2.5	62.5	25.0	12.5
	GGFRGRGGGRGTPRGRGGGRGGGRGGFRGG	50..79	3	5	‘F’: 2, ‘T’: 1, ‘P’: 1	9.5	2.3	60.0	26.7	13.3
>NP_989101.1 [Xenopus tropicalis]	PRGGRGGYGDRGGFGDRGGGRGRGGFRGRGGGGDRGGFGGRGGFGGRGGFGDRGGFRGGFKSPGRGGPRGGRGGRGG	7..83	2	15	‘P’: 3, ‘Y’: 1, ‘D’: 4, ‘F’: 7, ‘K’: 1, ‘S’: 1	23.7	2.5	55.8	22.1	22.1
>XP_003224982.1 [Anolis carolinensis]	PRGGRGDRGGRGGFGDRGRGGFRGGRGGGFNSPGRGGGPFRGGRGGSRGRGGPRGGGRGGRGGFRGG	7..73	3	14	‘P’: 4, ‘D’: 2, ‘F’: 5, ‘N’: 1, ‘S’: 2	21.5	2.1	53.7	25.4	20.9
>NP_032017.2 [Mus musculus]	PRGGGFGGRGGFGDRGGRGGGRGGRGGFGGGRGGFGGGGRGRGGGGGGFRGRGGGGGRGGGFQSGGNRGRGGGRGGKRG	7..85	4	12	‘P’: 1, ‘F’: 6, ‘D’: 1, ‘Q’: 1, ‘S’: 1, ‘N’: 1, ‘K’: 1	24.2	3.2	64.6	20.3	15.2
>NP_001427.2 [Homo sapiens]	PRGGGFGGRGGFGDRGGRGGRGGFGGGRGRGGGFRGRGRGGGGGGGGGGGGGRGGGGFHSGGNRGRGRGGKRG	7..79	6	9	‘P’: 1, ‘F’: 5, ‘D’: 1, ‘H’: 1, ‘S’: 1, ‘N’: 1, ‘K’: 1	22.7	3.1	64.4	20.6	15.1
NCL										
>NP_001070120.2 [Danio rerio]	ERGGGGRG	541..548	1	1	‘E’: 1	1.1	2.5	62.5	25.0	12.5
	GGRGGFGGGRGGFGGRGGGRGGFGGRGGGGRGGGFRGGRGGRGGGGGFRGGRGGGGRGG	633..691	0	12	‘F’: 5	8.4	3.5	71.2	20.3	8.5
>XP_031758857.1 [Xenopus tropicalis]	QRGGRGGFGRGGGFRGGRGGRGGGGGRGGFGGRGGGRGRGGFGGRGGGGFRGG	641..693	1	11	‘Q’: 1, ‘F’: 5	7.5	2.9	66.4	22.6	11.3
>XP_003225545.1 [Anolis carolinensis]	GQRGGGGGGFGRGGRGGGGRGGGRGGFGRGGGRGFGGRGGGFRGGRGG	634..681	1	9	‘Q’: 1, ‘F’: 4	6.9	3.3	68.8	20.8	10.4
>NP_035010.3 [Mus musculus]	GGRGGGRGGFGGRGGGRGGRGGFGGRGRGGFGGRGGFRGGRGG	651..693	1	9	‘F’: 4	6.1	2.9	67.4	23.3	9.3
>NP_005372.2 [Homo sapiens]	GGRGGGRGGFGGRGGGRGGRGGFGGRGRGGFGGRGGFRGGRGG	654..696	1	9	‘F’: 4	6.1	2.9	67.4	23.3	9.3
GAR1										
>NP_957269.2 [Danio rerio]	FRGGGGGRGGGFNRGGGGGRGGGFGGGRGGGFGGGRGGGFGGGRGGRGG	3..51	0	8	‘F’: 5, ‘N’: 1	21.8	4.4	71.4	16.3	12.2
	PRGGRGGGGRGGRGGGFRGGRGANGGGRGGFGGRGGGFGGRGGGGGGFRGGRGGGGGRGFRGG	162..224	2	11	‘P’: 1, ‘F’: 5, ‘A’: 1, ‘N’: 1	28.0	3.2	66.7	20.6	12.7
>NP_001011252.1 [Xenopus tropicalis]	FRGRGGFNRGGGGGRGGGGFGGRGGGRGGYGQGGGRGGFGRGGGRGGFNRGG	3..54	1	9	‘F’: 5, ‘N’: 2, ‘Y’: 1, ‘Q’: 1	23.9	3.3	63.5	19.2	17.3
	PRGGGRGGGRGGGRGRGGGRGGGGGFRGGRGGGFGGGGGFRGSRGGGFRGGRGFRGG	161..217	3	10	‘P’: 1, ‘F’: 5, ‘S’: 1	26.2	2.9	64.9	22.8	12.3
>XP_008110322.1 [Anolis carolinensis]	FRGRGGGNRGGGFNRGGGFNRGGGGFNRGGFSRGGGRGGFGRGGGRGGFNRGG	3..55	1	10	‘F’: 7, ‘N’: 5, ‘S’: 1	24.9	2.6	54.7	20.8	24.5
	PRGGRGGRGGRGGGRGGGGRGGGGFRGGRGGGGFRGGRGGGGGGRGFRGRG	162..212	3	10	‘P’: 1, ‘F’: 3	23.9	2.6	66.7	25.5	7.8
>NP_080854.1 [Mus musculus]	FRGGGRGGFNRGGGGGGFNRGGGSNNHFRGGGGGGGGSFRGGGGGGGGSFRGGGRGGFGRGGGRGG	3..68	0	10	‘F’: 7, ‘N’: 4, ‘S’: 3, ‘H’: 1	28.6	4.1	62.1	15.2	22.7
	PRGGGGGGRGGRGGGRGGGGRGGGRGGGFRGGRGGGGGFRGGRGGGGFRGRG	179..230	2	10	‘P’: 1, ‘F’: 3	22.5	3.0	69.2	23.1	7.7
>NP_061856.1 [Homo sapiens]	FRGGGRGGFNRGGGGGGFNRGGSSNHFRGGGGGGGGGNFRGGGRGGFGRGGGRGG	3..57	0	9	‘F’: 6, ‘N’: 4, ‘S’: 2, ‘H’: 1	25.4	3.7	60.0	16.4	23.6
	PRGGGRGGRGGGRGGGGRGGGRGGGFRGGRGGGGGGFRGGRGGGFRGRG	168..216	2	10	‘P’: 1, ‘F’: 3	22.6	2.8	67.4	24.5	8.2

**Table 3:** Analyses of GAR motifs in selected human RG/RGG-containing proteins by GMF.

Accession Number/Protein	Pattern	Position	RG	RGG	Else	Total%	G/R	G%	R%	Non-GR %
>NP_004951.1 FUS ^1^	DRGGRGRGG	212..220	1	2	‘D’: 1	1.7	1.7	55.6	33.3	11.1
	PRGRGGGRGGRGGMGGSDRGG	241..261	1	4	‘P’: 1, ‘M’: 1, ‘S’: 1, ‘D’: 1	4.0	2.4	57.1	23.8	19.1
	NRGGGNGRGGRGRGGPMGRGG	376..396	1	4	‘N’: 2, ‘P’: 1, ‘M’: 1	4.0	2.4	57.1	23.8	19.1
	RRGGRGGYDRGGYRGRGGDRGGFRGGRGGGDRGG	472..505	1	8	‘Y’: 2, ‘D’: 3, ‘F’: 1	6.5	1.8	52.9	29.4	17.7
>sp|Q01844.1 EWS	NRGRGRGGFDRGGMSRGGRGGGRGGMGSAGERGG	299..332	2	6	‘N’: 1, ‘F’: 1, ‘D’: 1, ‘M’: 2, ‘S’: 2, ‘A’: 1, ‘E’: 1	5.2	2.1	50.0	23.5	26.5
	MRGGLPPREGRGMPPPLRGG	454..473	1	2	‘M’: 2, ‘L’: 2, ‘P’: 5, ‘E’: 1	3.1	1.5	30.0	20.0	50.0
	GGRGGDRGGFPPRGPRGSRG	488..507	3	2	‘D’: 1, ‘F’: 1, ‘P’: 3, ‘S’: 1	3.1	1.8	45.0	25.0	30.0
	GGDRGRGGPGGMRGGRGGLMDRGGPGGMFRGGRGGDRGGFRGGRGMDRGGFGGGRRGG	560..617	2	10	‘D’: 4, ‘P’: 2, ‘M’: 4, ‘L’: 1, ‘F’: 3	8.8	2.4	53.5	22.4	24.1
	GGRRGGRGG	630..638	0	2		1.4	2.0	66.7	33.3	0.0
>NP_631961.1 TAF15	NRGYGGSQGGGRGRGGYDKDGRG	174..196	3	1	‘N’: 1, ‘Y’: 2, ‘S’: 1, ‘Q’: 1, ‘D’: 2, ‘K’: 1	3.9	2.8	47.8	17.4	34.8
	MRGGGSGGGRRGRGGYRGRGGFQGRGG	325..351	2	4	‘M’: 1, ‘S’: 1, ‘Y’: 1, ‘F’: 1, ‘Q’: 1	4.6	2.1	55.6	25.9	18.5
	FRGRGYGGERGYRGRGGRGGDRGG	394..417	4	3	‘F’: 1, ‘Y’: 2, ‘E’: 1, ‘D’: 1	4.1	1.7	50.0	29.2	20.8
	GGDRGGGYGGDRGGGYGGDRGGGYGGDRGGYGGDRGGGYGGDRGGYGGDRGGYGGDRGGYGGDRGGYGGDRSRGGYGGDRGG	456..537	0	11	‘D’: 11, ‘Y’: 10, ‘S’: 1	13.9	4.0	58.5	14.6	26.8
	GGDRGGGYGGDRGG	559..572	0	2	‘D’: 2, ‘Y’: 1	2.4	4.5	64.3	14.3	21.4
>sp|Q8ND56.3 Lsm14a (RAP55A)	RRGRGGHRGGRG	269..280	2	2	‘H’: 1	2.6	1.2	50.0	41.7	8.3
	NRGRGGYRGRGGLGFRGGRGRGGGRGGTFTAPRGFRGGFRGGRGG	403..447	4	8	‘N’: 1, ‘Y’: 1, ‘L’: 1, ‘F’: 4, ‘T’: 2, ‘A’: 1, ‘P’: 1	9.7	1.8	48.9	26.7	24.4
>NP_001018077.1 SERBP1	IRGRGGLGRGRGGRGRGMGRGDGFDSRG	162..189	6	2	‘I’: 1, ‘L’: 1, ‘M’: 1, ‘D’: 2, ‘F’: 1, ‘S’: 1	6.9	1.6	46.4	28.6	25.0
	GRGGRGGRGGRGRGGRPNRG	366..385	2	4	‘P’: 1, ‘N’: 1	4.9	1.6	55.0	35.0	10.0
>NP_112420.1 hnRNPA1	DRGSGKKRG	139..147	2	0	‘D’: 1, ‘S’: 1, ‘K’: 2	2.4	1.5	33.3	22.2	44.4
	GRGGNFSGRGGFGGSRGG	217..234	0	3	‘N’: 1, ‘F’: 2, ‘S’: 2	4.8	3.3	55.6	16.7	27.8
>NP_114032.2 hnRNPU	NRGGGHRGRGGFNMRGGNFRGGAPGNRGGYNRRGNMPQRGG	701..741	2	6	‘N’: 6, ‘H’: 1, ‘F’: 2, ‘M’: 2, ‘A’: 1, ‘P’: 2, ‘Y’: 1, ‘Q’: 1	5.0	1.8	39.0	22.0	39.0
	GRGSYSNRGNYNRGGMPNRGNYNQNFRGRGNNRG	761..794	6	1	‘S’: 2, ‘Y’: 3, ‘N’: 9, ‘M’: 1, ‘P’: 1, ‘Q’: 1, ‘F’: 1	4.1	1.3	26.5	20.6	52.9
>NP_077726.1 DDX4	NRGFSKRGG	124..132	1	1	‘N’: 1, ‘F’: 1, ‘S’: 1, ‘K’: 1	1.2	1.5	33.3	22.2	44.4
	RRGGRGSFRGCRGG	146..159	2	2	‘S’: 1, ‘F’: 1, ‘C’: 1	1.9	1.2	42.9	35.7	21.4
>NP_005745.1 G3BP1	LRGPGGPRGGLGGGMRGPPRGG	428..449	2	2	‘L’: 2, ‘P’: 4, ‘M’: 1	4.7	2.8	50.0	18.2	31.8
>NP_002015.1 FMRP	GRGSRPYRNRGHGRRG	470..485	3	0	‘S’: 1, ‘P’: 1, ‘Y’: 1, ‘N’: 1, ‘H’: 1	2.5	0.8	31.3	37.5	31.3
	RRGDGRRRGGGGRGQGGRGRGG	527..548	3	2	‘D’: 1, ‘Q’: 1	3.5	1.5	54.6	36.4	9.1
>NP_005078.2 FXR1	GRGRGRRG	385..392	3	0		1.3	1.0	50.0	50.0	0.0
	GGRGRSVSGGRGRGGPRGG	443..461	2	2	‘S’: 2, ‘V’: 1, ‘P’: 1	3.1	2.0	52.6	26.3	21.1
>NP_004851.2 FXR2	GGRGRG	430..435	2	0		0.9	2.0	66.7	33.3	0.0
	GGRGRG	486..491	2	0		0.9	2.0	66.7	33.3	0.0
>NP_006550.1 KHDR1 (SAM68)	SRGGGGGSRGG	44..54	0	2	‘S’: 2	2.5	3.5	63.6	18.2	18.2
	SRGRGVPVRGRG	281..292	4	0	‘S’: 1, ‘V’: 2, ‘P’: 1	2.7	1.0	33.3	33.3	33.3
	PRGRGVGPPRGALVRGTPVRGAITRGATVTRG	301..332	7	0	‘P’: 4, ‘V’: 4, ‘A’: 3, ‘L’: 1, ‘T’: 4, ‘I’: 1	7.2	1.1	25.0	21.9	53.1
>NP_005889.3 Caprin-1	SRGVSRGGSRGARGLMNGYRGPANGFRGG	607..635	4	2	‘S’: 3, ‘V’: 1, ‘A’: 2, ‘L’: 1, ‘M’: 1, ‘N’: 2, ‘Y’: 1, ‘P’: 1, ‘F’: 1	4.1	1.7	34.5	20.7	44.8
	KRGSGQSGPRGAPRGRGGPPRPNRG	675..699	4	1	‘K’: 1, ‘S’: 2, ‘Q’: 1, ‘P’: 5, ‘A’: 1, ‘N’: 1	3.5	1.3	32.0	24.0	44.0
>NP_056422.2 CHTOP	ARGAIGGRGLPIIQRGLPRGGLRGG	96..120	3	2	‘A’: 2, ‘I’: 3, ‘L’: 3, ‘P’: 2, ‘Q’: 1	10.1	1.8	36.0	20.0	44.0
	LRGGMSLRGQNLLRGG	127..142	1	2	‘L’: 4, ‘M’: 1, ‘S’: 1, ‘Q’: 1, ‘N’: 1	6.5	1.7	31.3	18.8	50.0
	RRGGVRGRGGPGRGGLGRGAMGRGGIGGRGRGMIGRGRGGFGGRGRGRGRGRG	152..204	10	5	‘V’: 1, ‘P’: 1, ‘L’: 1, ‘A’: 1, ‘M’: 2, ‘I’: 2, ‘F’: 1	21.4	1.8	52.8	30.2	17.0
>NP_055542.1 kmt2b (MML4)	ARGRFPGRPRGAGGGGGRGGRG	16..37	3	1	‘A’: 2, ‘F’: 1, ‘P’: 2	0.8	1.8	50.0	27.3	22.7
	QRGRGRGRGRGWGPSRG	90..106	6	0	‘Q’: 1, ‘W’: 1, ‘P’: 1, ‘S’: 1	0.6	1.2	41.2	35.3	23.5
	QRGRAPRGRG	144..153	3	0	‘Q’: 1, ‘A’: 1, ‘P’: 1	0.4	0.8	30.0	40.0	30.0
	RRGGQSSRGGRGGRGRGRGG	280..299	2	4	‘Q’: 1, ‘S’: 2	0.7	1.4	50.0	35.0	15.0

^1^ The protein names labeled in yellow belong to tri-RGG, those in green belong to tri-RG, those in blue belong to di-RGG, and those in grey belong to di-RG, as classified by Thandapani et al. [8].

**Table 4:** Analyses of GAR motifs with the number of RG plus RGG repeats of more than 10 in human proteome by GMF.

Accession Number/Name ^1^	Pattern	Position	RG	RGG	Else	%	G/R	G%	R%	Non-GR %	Isoforms
>NP_055983.1/ ZC3H4	SRGRGSRGRGRGYRGRGSRGGSRGRGMGRGSRGRGRG	235..271	13	1	‘S’: 5, ‘Y’: 1, ‘M’: 1	2.8	1.1	43.2	37.8	18.9	11
	SRGRGLSRGRGRGSRGRGKGMGRGRGRGGSRGG	319..351	9	2	‘S’: 4, ‘L’: 1, ‘K’: 1, ‘M’: 1	2.5	1.4	45.5	33.3	21.2	11
>NP_003918.1/ MBD2	GARGGGRGRGRWKQAGRGGGVCGRGRGRGRGRGRGRGRGRGRG	53..95	12	2	‘A’: 2, ‘W’: 1, ‘K’: 1, ‘Q’: 1, ‘V’: 1, ‘C’: 1	10.5	1.4	48.8	34.9	16.3	2
>NP_001095868.1/ hnRNP R	VRGRGGGRGGRGAPPPPRGRGAPPPRGRAGYSQRGAPLGPPRGSRGGRGGPAQQQRGRGSRGSRGNRGG	502..570	11	5	‘V’: 1, ‘A’: 5, ‘P’: 11, ‘Y’: 1, ‘S’: 4, ‘Q’: 4, ‘L’: 1, ‘N’: 1	10.8	1.4	34.8	24.6	40.6	17
>NP_006363.4 /hnRNP Q isoform 1	GARGRGGRGARGAAPSRGRGAAPPRGRAGYSQRGGPGSARGVRGARGGAQQQRGRGVRGARGGRGG	494..559	11	5	‘A’: 11, ‘P’: 4, ‘S’: 3, ‘Y’: 1, ‘Q’: 4, ‘V’: 2	10.6	1.4	36.4	25.8	37.9	6
>NP_001153145.1/ hnRNP Q isoform 2	GARGRGGRGARGAAPSRGRGAAPPRGRAGYSQRGGPGSARGVRGARGGAQQQRGRG	396..451	10	3	‘A’: 10, ‘P’: 4, ‘S’: 3, ‘Y’: 1, ‘Q’: 4, ‘V’: 1	12.1	1.4	33.9	25.0	41.1	5
>NP_001153148.1/ hnRNP Q isoform 5	GARGRGGRGARGAAPSRGRGAAPPRGRAGYSQRGGPGSARGVRGARGGAQQQRGRGG	494..550	9	4	‘A’: 10, ‘P’: 4, ‘S’: 3, ‘Y’: 1, ‘Q’: 4, ‘V’: 1	10.2	1.4	35.1	24.6	40.4	4
>NP_003741.1/ eIF-3A	DRGPRRGLDDDRGPRRGMDDDRGPRRGMDDDRGPRRGMDDDRGPRRGLDDDRG	1066..1118	11	0	‘D’: 16, ‘P’: 5, ‘L’: 2, ‘M’: 3	3.8	0.7	20.8	30.2	49.1	1
>NP_001296171.1/myosin XVB	GRGHGRGSKGRGRGKADEGRGHERGDEGRGRGKADEGRGHERGYEGRG	414..461	11	0	‘H’: 3, ‘S’: 1, ‘K’: 3, ‘A’: 2, ‘D’: 3, ‘E’: 6, ‘Y’: 1	1.6	1.6	37.5	22.9	39.6	1
>NP_056422.2/ CHTOP	RRGGVRGRGGPGRGGLGRGAMGRGGIGGRGRGMIGRGRGGFGGRGRGRGRGRG	152..204	10	5	‘V’: 1, ‘P’: 1, ‘L’: 1, ‘A’: 1, ‘M’: 2, ‘I’: 2, ‘F’: 1	21.4	1.8	52.8	30.2	17.0	2
>NP_001273560.1/RBM26	KRGILSSGRGRGIHSRGRGAVHGRGRGRGRGRG	849..881	10	0	‘K’: 1, ‘I’: 2, ‘L’: 1, ‘S’: 3, ‘H’: 2, ‘A’: 1, ‘V’: 1	3.3	1.2	36.4	30.3	33.3	40
>NP_694984.5/ BRWD3	SRGGRGRGGRGRGSRGRGGGGTRGRGRGRGGRGASRG	1683..1719	9	4	‘S’: 3, ‘T’: 1, ‘A’: 1	2.1	1.5	51.4	35.1	13.5	3
>NP_001276342.1/EHMT2	MRGLPRGRGLMRARGRGRAAPPGSRGRGRGGPHRGRG	1..37	9	1	‘M’: 2, ‘L’: 2, ‘P’: 4, ‘A’: 3, ‘S’: 1, ‘H’: 1	3.0	1.0	32.4	32.4	35.1	4
>NP_689813.2/ ZNF579	HRGRGRGRGRGRGRGRGRGRGG	15..36	9	1	‘H’: 1	3.9	1.1	50.0	45.5	4.5	4
>NP_002943.2/ RPS2	GNRGGFRGGFGSGIRGRGRGRGRGRGRGRGARGG	20..53	8	3	‘N’: 1, ‘F’: 2, ‘S’: 1, ‘I’: 1, ‘A’: 1	11.6	1.5	50.0	32.4	17.6	1
>NP_003478.1/ TAF15	GGDRGGGYGGDRGGGYGGDRGGGYGGDRGGYGGDRGGGYGGDRGGYGGDRGGYGGDRGGYGGDRGGYGGDRSRGGYGGDRGG	453..534	0	11	‘D’: 11, ‘Y’: 10, ‘S’: 1	13.9	4.0	58.5	14.6	26.8	3
>NP_053733.2/ EWS	GGDRGRGGPGGMRGGRGGLMDRGGPGGMFRGGRGGDRGGFRGGRGMDRGGFGGGRRGG	565..622	2	10	‘D’: 4, ‘P’: 2, ‘M’: 4, ‘L’: 1, ‘F’: 3	8.8	2.4	53.4	22.4	24.1	28
>NP_061856.1/ GAR1	PRGGGRGGRGGGRGGGGRGGGRGGGFRGGRGGGGGGFRGGRGGGFRGRG	168..216	2	10	‘P’: 1, ‘F’: 3	22.6	2.8	67.3	24.5	8.2	2
>NP_001427.2/ fibrillarin	PRGGGFGGRGGFGDRGGRGGRGGFGGGRGRGGGFRGRGRGGGGGGGGGGGGGRGGGGFHSGGNRGRGRGGKRG	7..79	6	9	‘P’: 1, ‘F’: 5, ‘D’: 1, ‘H’: 1, ‘S’: 1, ‘N’: 1, ‘K’: 1	22.7	3.1	64.4	20.5	15.1	2
>NP_005372.2/ nucleolin	GGRGGGRGGFGGRGGGRGGRGGFGGRGRGGFGGRGGFRGGRGG	654..696	1	9	‘F’: 4	6.1	2.9	67.4	23.3	9.3	1
>NP_001107565.1/LSM14	NRGRGGYRGRGGLGFRGGRGRGGGRGGTFTAPRGFRGGFRGGRGG	403..447	4	8	‘N’: 1, ‘Y’: 1, ‘L’: 1, ‘F’: 4, ‘T’: 2, ‘A’: 1, ‘P’: 1	9.7	1.8	48.9	26.7	24.4	24

^1^ The protein name is the short common name of the protein, not necessarily the same as that following the accession number as shown the Supplementary Table S3.

## Data Availability

The data presented in this study are available in Supplementary Materials.

## Supplementary Materials

The following supporting information can be downloaded at: https://www.mdpi.com/article/10.3390/genes14020330/s1, Supplementary Figure S1. Alignment of the sequences in the N-terminal GAR domain of FBL; Supplementary Table S1. Direct .csv output of the analyses of FBL, NCL and GAR1 of five model vertebrate species by GMF; Supplementary Table S2. Direct .csv output of the analyses of selected human RG/RGG-containing proteins by GMF; Supplementary Table S3. .csv output of the analyses by GMF the isoforms and all motifs in the proteins containing GAR motifs RG plus RGG repeats of more than 10 in human proteome; Supplementary File S1. Direct .txt output of the analyses of FBL, NCL, and GAR1 of five model vertebrate species by GMF; Supplementary File S2. Direct .txt output of the analyses of selected human RG/RGG-containing proteins by GMF.

## References

[1] Shubina M.Y., Arifulin E.A., Sorokin D.V., Sosina M.A., Tikhomirova M.A., Serebryakova M.V., Smirnova T., Sokolov S.S., Musinova Y.R., Sheval E.V. (2020). The GAR domain integrates functions that are necessary for the proper localization of fibrillarin (FBL) inside eukaryotic cells. PeerJ.

[2] Tollervey D., Lehtonen H., Jansen R., Kern H., Hurt E.C. (1993). Temperature-sensitive mutations demonstrate roles for yeast fibrillarin in pre-rRNA processing, pre-rRNA methylation, and ribosome assembly. Cell.

[3] Snaar S., Wiesmeijer K., Jochemsen A.G., Tanke H.J., Dirks R.W. (2000). Mutational analysis of fibrillarin and its mobility in living human cells. J. Cell Biol..

[4] El Hassouni B., Sarkisjan D., Vos J.C., Giovannetti E., Peters G.J. (2019). Targeting the Ribosome Biogenesis Key Molecule Fibrillarin to Avoid Chemoresistance. Curr. Med. Chem..

[5] Stamm S., Lodmell J.S. (2019). C/D box snoRNAs in viral infections: RNA viruses use old dogs for new tricks. Non-Coding RNA Res..

[6] Feric M., Vaidya N., Harmon T.S., Mitrea D.M., Zhu L., Richardson T.M., Kriwacki R.W., Pappu R.V., Brangwynne C.P. (2016). Coexisting Liquid Phases Underlie Nucleolar Subcompartments. Cell.

[7] Yao R.W., Xu G., Wang Y., Shan L., Luan P.F., Wang Y., Wu M., Yang L.Z., Xing Y.H., Yang L. (2019). Nascent Pre-rRNA Sorting via Phase Separation Drives the Assembly of Dense Fibrillar Components in the Human Nucleolus. Mol. Cell.

[8] Thandapani P., O’Connor T.R., Bailey T.L., Richard S. (2013). Defining the RGG/RG motif. Mol. Cell.

[9] Dragon F., Pogacić V., Filipowicz W. (2000). In vitro assembly of human H/ACA small nucleolar RNPs reveals unique features of U17 and telomerase RNAs. Mol. Cell Biol..

[10] Lischwe M.A., Ochs R.L., Reddy R., Cook R.G., Yeoman L.C., Tan E.M., Reichlin M., Busch H. (1985). Purification and partial characterization of a nucleolar scleroderma antigen (Mr = 34,000; pI, 8.5) rich in NG,NG-dimethylarginine. J. Biol. Chem..

[11] Lischwe M.A., Roberts K.D., Yeoman L.C., Busch H. (1982). Nucleolar specific acidic phosphoprotein C23 is highly methylated. J. Biol. Chem..

[12] Frankel A., Clarke S. (1999). RNase treatment of yeast and mammalian cell extracts affects in vitro substrate methylation by type I protein arginine N-methyltransferases. Biochem. Biophys. Res. Commun..

[13] Whitehead S.E., Jones K.W., Zhang X., Cheng X., Terns R.M., Terns M.P. (2002). Determinants of the interaction of the spinal muscular atrophy disease protein SMN with the dimethylarginine-modified box H/ACA small nucleolar ribonucleoprotein GAR1. J. Biol. Chem..

[14] Kiledjian M., Dreyfuss G. (1992). Primary structure and binding activity of the hnRNP U protein: Binding RNA through RGG box. Embo. J..

[15] Liu Q., Dreyfuss G. (1995). In vivo and in vitro arginine methylation of RNA-binding proteins. Mol. Cell Biol..

[16] Bedford M.T., Clarke S.G. (2009). Protein Arginine Methylation in Mammals: Who, What, and Why. Mol. Cell.

[17] Blanc R.S., Richard S. (2017). Arginine Methylation: The Coming of Age. Mol. Cell.

[18] Smith D.L., Erce M.A., Lai Y.W., Tomasetig F., Hart-Smith G., Hamey J.J., Wilkins M.R. (2020). Crosstalk of Phosphorylation and Arginine Methylation in Disordered SRGG Repeats of Saccharomycescerevisiae Fibrillarin and Its Association with Nucleolar Localization. J. Mol. Biol..

[19] Hofweber M., Dormann D. (2019). Friend or foe-Post-translational modifications as regulators of phase separation and RNP granule dynamics. J. Biol. Chem..

[20] Ai L.S., Lin C.H., Hsieh M., Li C. (1999). Arginine methylation of a glycine and arginine rich peptide derived from sequences of human FMRP and fibrillarin. Proc. Natl. Sci. Counc. Repub. China B.

[21] Lin C.H., Huang H.M., Hsieh M., Pollard K.M., Li C. (2002). Arginine methylation of recombinant murine fibrillarin by protein arginine methyltransferase. J. Protein Chem..

[22] Lee Y.J., Hsieh W.Y., Chen L.Y., Li C. (2012). Protein arginine methylation of SERBP1 by protein arginine methyltransferase 1 affects cytoplasmic/nuclear distribution. J. Cell Biochem..

[23] Lee Y.J., Wei H.M., Chen L.Y., Li C. (2014). Localization of SERBP1 in stress granules and nucleoli. FEBS J..

[24] Wei H.M., Hu H.H., Chang G.Y., Lee Y.J., Li Y.C., Chang H.H., Li C. (2014). Arginine methylation of the cellular nucleic acid binding protein does not affect its subcellular localization but impedes RNA binding. FEBS Lett..

[25] Chong P.A., Vernon R.M., Forman-Kay J.D. (2018). RGG/RG Motif Regions in RNA Binding and Phase Separation. J. Mol. Biol..

[26] Li K.K.C., Chau B.L., Lee K.A.W. (2018). Differential interaction of PRMT1 with RGG-boxes of the FET family proteins EWS and TAF15. Protein Sci..

[27] Bikkavilli R.K., Malbon C.C. (2011). Arginine methylation of G3BP1 in response to Wnt3a regulates -catenin mRNA. J. Cell Sci..

[28] Guo Z., Zheng L., Xu H., Dai H., Zhou M., Pascua M.R., Chen Q.M., Shen B. (2010). Methylation of FEN1 suppresses nearby phosphorylation and facilitates PCNA binding. Nat. Chem. Biol..

[29] Angrand G., Quillevere A., Loaec N., Dinh V.T., Le Senechal R., Chennoufi R., Duchambon P., Keruzore M., Martins R.P., Teulade-Fichou M.P. (2022). Type I arginine methyltransferases are intervention points to unveil the oncogenic Epstein-Barr virus to the immune system. Nucleic Acids Res..

[30] Campbell M., Chang P.C., Huerta S., Izumiya C., Davis R., Tepper C.G., Kim K.Y., Shevchenko B., Wang D.H., Jung J.U. (2012). Protein arginine methyltransferase 1-directed methylation of Kaposi sarcoma-associated herpesvirus latency-associated nuclear antigen. J. Biol. Chem..

[31] Mostaqul Huq M.D., Gupta P., Tsai N.-P., White R., Parker M.G., Wei L.-N. (2006). Suppression of receptor interacting protein 140 repressive activity by protein arginine methylation. EMBO J..

[32] Masuzawa T., Oyoshi T. (2020). Roles of the RGG Domain and RNA Recognition Motif of Nucleolin in G-Quadruplex Stabilization. ACS Omega.

[33] Guo A., Gu H., Zhou J., Mulhern D., Wang Y., Lee K.A., Yang V., Aguiar M., Kornhauser J., Jia X. (2014). Immunoaffinity enrichment and mass spectrometry analysis of protein methylation. Mol. Cell Proteom..

[34] Qamar S., Wang G., Randle S.J., Ruggeri F.S., Varela J.A., Lin J.Q., Phillips E.C., Miyashita A., Williams D., Strohl F. (2018). FUS Phase Separation Is Modulated by a Molecular Chaperone and Methylation of Arginine Cation-pi Interactions. Cell.

[35] Kaneb H.M., Dion P.A., Rouleau G.A. (2012). The FUS about arginine methylation in ALS and FTLD. Embo J..

[36] Weimann M., Grossmann A., Woodsmith J., Özkan Z., Birth P., Meierhofer D., Benlasfer N., Valovka T., Timmermann B., Wanker E.E. (2013). A Y2H-seq approach defines the human protein methyltransferase interactome. Nat. Methods.

[37] Gittings L.M., Foti S.C., Benson B.C., Gami-Patel P., Isaacs A.M., Lashley T. (2019). Heterogeneous nuclear ribonucleoproteins R and Q accumulate in pathological inclusions in FTLD-FUS. Acta Neuropathol. Commun..

[38] Estell C., Davidson L., Steketee P.C., Monier A., West S. (2021). ZC3H4 restricts non-coding transcription in human cells. eLife.

[39] Musiani D., Bok J., Massignani E., Wu L., Tabaglio T., Ippolito M.R., Cuomo A., Ozbek U., Zorgati H., Ghoshdastider U. (2019). Proteomics profiling of arginine methylation defines PRMT5 substrate specificity. Sci. Signal.

[40] van Dijk T.B., Gillemans N., Stein C., Fanis P., Demmers J., van de Corput M., Essers J., Grosveld F., Bauer U.M., Philipsen S. (2010). Friend of Prmt1, a novel chromatin target of protein arginine methyltransferases. Mol. Cell Biol..

[41] Takai H., Masuda K., Sato T., Sakaguchi Y., Suzuki T., Suzuki T., Koyama-Nasu R., Nasu-Nishimura Y., Katou Y., Ogawa H. (2014). 5-Hydroxymethylcytosine plays a critical role in glioblastomagenesis by recruiting the CHTOP-methylosome complex. Cell Rep..

[42] Ng H.H., Zhang Y., Hendrich B., Johnson C.A., Turner B.M., Erdjument-Bromage H., Tempst P., Reinberg D., Bird A. (1999). MBD2 is a transcriptional repressor belonging to the MeCP1 histone deacetylase complex. Nat. Genet..

[43] Tachibana M., Sugimoto K., Fukushima T., Shinkai Y. (2001). Set domain-containing protein, G9a, is a novel lysine-preferring mammalian histone methyltransferase with hyperactivity and specific selectivity to lysines 9 and 27 of histone H3. J. Biol. Chem..

[44] Rippe K. (2022). Liquid-Liquid Phase Separation in Chromatin. Cold Spring Harb. Perspect. Biol..

[45] Greig J.A., Nguyen T.A., Lee M., Holehouse A.S., Posey A.E., Pappu R.V., Jedd G. (2020). Arginine-Enriched Mixed-Charge Domains Provide Cohesion for Nuclear Speckle Condensation. Mol. Cell.

[46] Swiercz R., Person M.D., Bedford M.T. (2005). Ribosomal protein S2 is a substrate for mammalian PRMT3 (protein arginine methyltransferase 3). Biochem. J..

[47] Swiercz R., Cheng D., Kim D., Bedford M.T. (2007). Ribosomal protein rpS2 is hypomethylated in PRMT3-deficient mice. J. Biol. Chem..

