# Supplementary material for: Identification and Characterization of Glycine- and Arginine-Rich Motifs in Proteins by a Novel GAR Motif Finder Program
Source: Genes (Basel). 2023 Jan 27;14(2):330. doi: 10.3390/genes14020330 (PMC9957100; doi:10.3390/genes14020330)
Supplement: Supplementary file 1 [file genes-14-00330-s001.zip › Supplementary Figure S1.pdf]

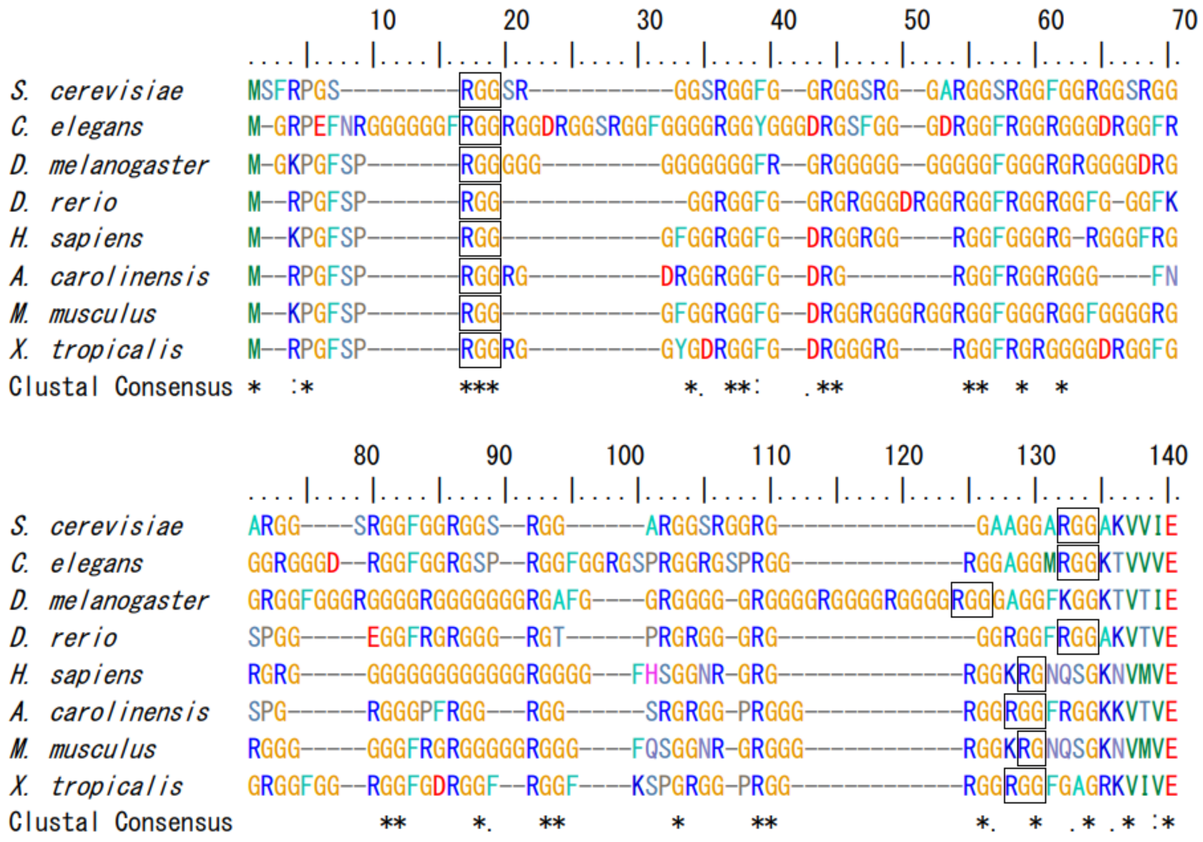

Supplementary Figure S1. Alignment of the sequences in the N-terminal GAR domain of FBL. The FBL sequences in yeast (*S. cerevisiae)*, nematodes (*C. elegans*) and fruit fly (*D. melanogaster*) and 5 vertebrate species (*D. rerio*, *X. tropicalis*, *A. carolinensis*, *H. sapiens* and *M. musculus*) are aligned and the lignments of the GAR domains are shown. The alignment was conducted by ClustalW with manual adjustments. The start and end RG(G) of the GAR domains are boxed.
